# Poster Session II A330 ANALYSIS OF ESOPHAGEAL CHARACTERISTICS IN CHILDREN WITH ESOPHAGEAL ATRESIA USING FLIP PANOMETRY

**DOI:** 10.1093/jcag/gwaf042.330

**Published:** 2026-02-13

**Authors:** M Awouters, C Faure

**Affiliations:** Pediatric gastroenterology, Centre Hospitalier Universitaire Sainte-Justine, Montreal, QC, Canada; Pediatric gastroenterology, Centre Hospitalier Universitaire Sainte-Justine, Montreal, QC, Canada

## Abstract

**Background:**

Esophageal atresia (EA) is the most common congenital abnormality of the oesophagus and is associated with long-term complications including dysmotility, dysphagia, gastroesophageal reflux disease, and eosinophilic oesophagitis (EoE).

**Aims:**

To assess esophageal mechanical and functional properties in children with EA and to evaluate the utility of Functional Lumen Imaging Probe (FLIP) panometry in this patient population.

**Methods:**

We retrospectively analysed FLIP panometry studies performed during endoscopic follow-up in EA patients. An 8- or 16-cm balloon was selected according to patient height. Where possible, we applied the 2025 Dallas criteria for data analysis. Measurements included esophagogastric junction (EGJ) characteristics, distensibility plateau (DP), compliance over 8cm and secondary peristalsis.

**Results:**

Thirty-two children with EA were included, with a median age of 7,4 years (9 months – 17 years). DP corresponded with the anastomosis in most patients, with a median DP of 14mm and a distensibility index (DI) at DP of 4,0mm^2/mmHg. Both DP and DI at DP correlated with age and height. A DP of less than 15mm was associated with food impaction, while DI at DP was correlated with both dysphagia and food impaction.

Secondary peristalsis was absent in 34%, diminished in 22%, normal in 28%, disordered/impaired in 13%, and spastic in 3%. A trend towards reflux oesophagitis was observed in patients with absent or diminished peristalsis. An EGJ DI of more than 4mm^2/mmHg significantly increased esophagitis risk.

Reduced esophageal compliance was found in 37,5%, but was not significantly correlated with age nor DP. EoE was correlated with reduced compliance, but not with secondary peristalsis or anastomosis characteristics. The median maximal pressure during the procedure was 70mmHg. Maximal filling volume was reached in only 41% of patients.

Three infants (≤ 2 years old) with severe tracheomalacia developed mild to moderate respiratory problems during balloon filling due to external trachea compression. No other complications occurred.

**Conclusions:**

FLIP panometry is feasible in children with EA from a young age and provides valuable insights into the anastomosis characteristics, esophageal anatomy and motility. Caution is warranted in infants with severe tracheomalacia.

**Funding Agencies:**

None

